# Supplementary material for: Computational Prediction and Structural Analysis of α-Hairpinins, a Ubiquitous Family of Antimicrobial Peptides, Using the Cysmotif Searcher Pipeline
Source: Antibiotics (Basel). 2024 Oct 30;13(11):1019. doi: 10.3390/antibiotics13111019 (PMC11591084; doi:10.3390/antibiotics13111019)
Supplement: Supplementary file 1 [file antibiotics-13-01019-s001.zip › Supplementary_Figures.pdf]

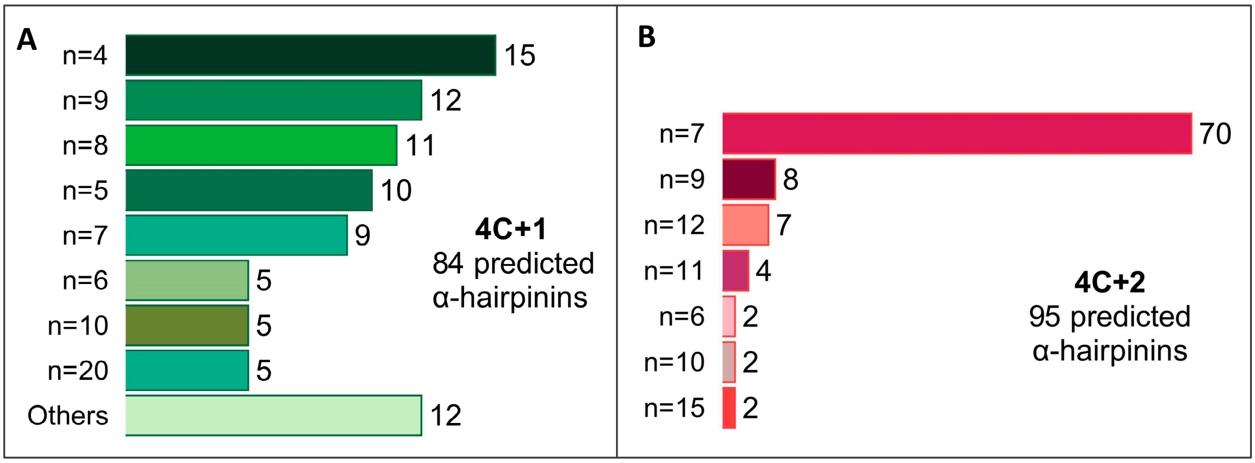

Figure S1. A bar chart displaying a distribution of different X values found in predicted peptides with 4C+1 and 4C+2 motifs. (**A**) 4C+1 motif, $C^5X_{\geq4}C^1X_3C^2X_{4\text{-}20}C^3X_3C^4$; (**B**) 4C+2 motif, $C^6XC^5X_{\geq4}C^1X_3C^2X_{4\text{-}20}C^3X_3C^4$.

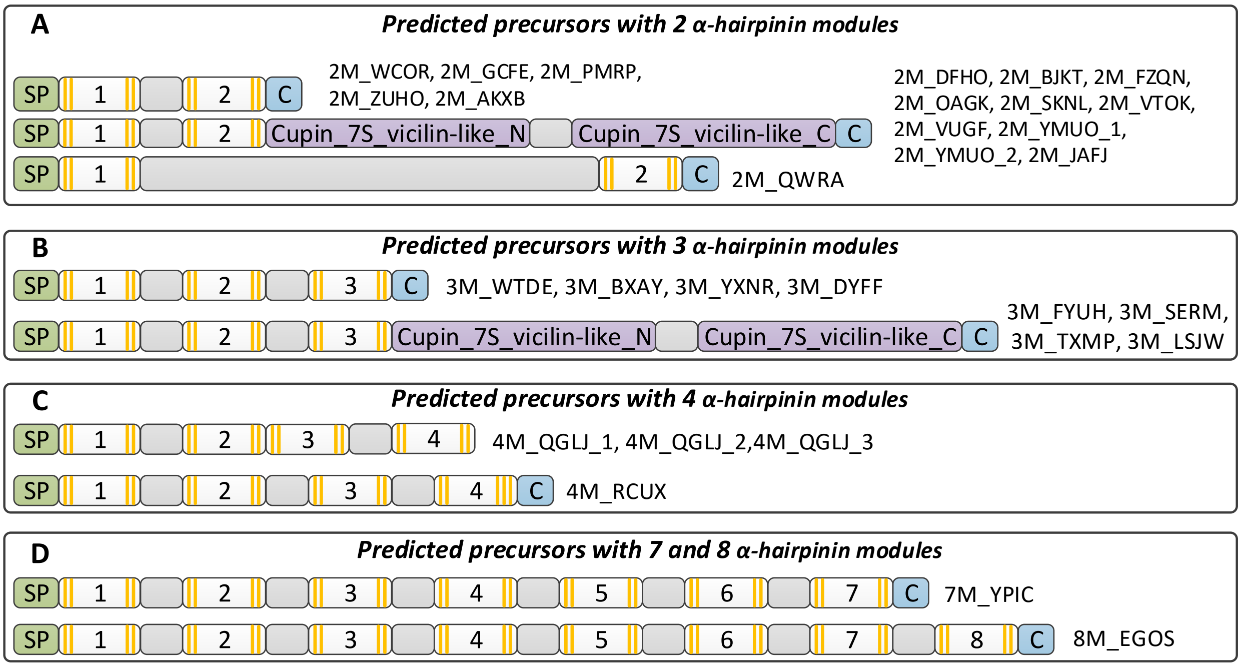

Figure S2. Structure of predicted modular α-hairpinin precursors. (A) Predicted precursors with 2 α-hairpinin modules. (B) Predicted precursors with 3 α-hairpinin modules. (C) Predicted precursors with 4 α-hairpinin modules. (D) Predicted precursors with 7 and 8 α-hairpinin modules. Signal peptides are shown as green boxes; α-hairpinin modules are displayed as white boxes with cysteine residues designated as orange sticks; vicilin-like domains are shown as violet boxes; spacers and regions with unknown functions are displayed as grey boxes; C-terminal domains are shown as blue boxes. Transcriptome accession codes are indicated for each sequence.

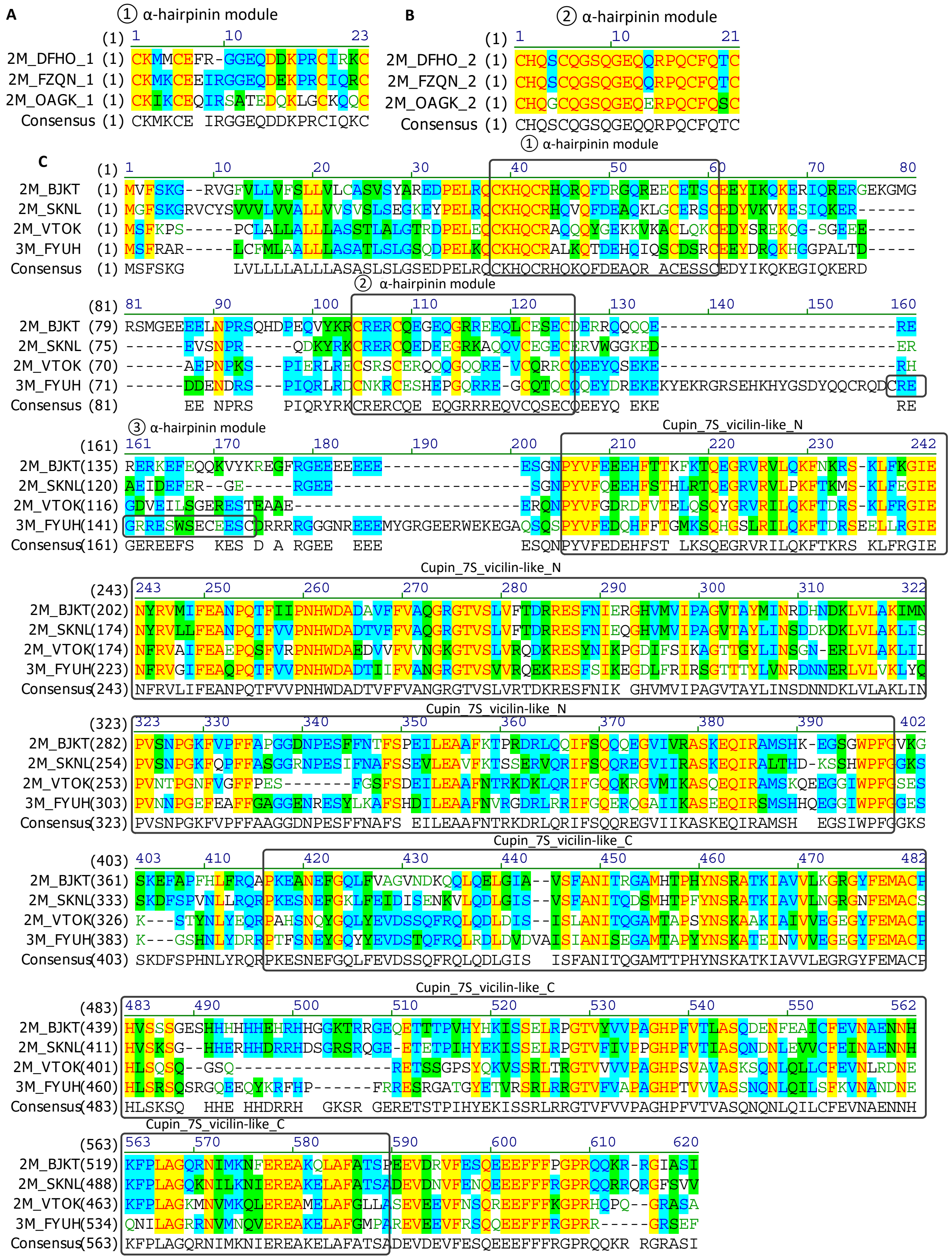

Figure S3. Multiple alignments of α-hairpinin-modules referred to the first class of precursors. (**A**), (**B**) Multiple alignment of the first and the second α-hairpinin module. (**C**) Multiple alignment of the whole α-

hairpinin precursors. Designation of modules: the first number and letter terms the number of modules in the precursor, 4 letter code is 1KP abbreviation of plant and the last number names the order of the module. DFHO, *Danaea nodosa* (a fern, Marattiaceae family); FZQN, *Silene latifolia* (Caryophyllaceae); OAGK, *Matricaria matricarioides* (Asteraceae); SKNL, *Saponaria officinalis* (Caryophyllaceae); BJKT; *Delosperma echinatum* (Aizoaceae, a secculent); VTOK, *Orobanche fasciculate* (Orobanchaceae, parasitic); VUGF, *Punica granatum* (Lythraceae); FYUH, *Lavandula angustifolia* (Lamiaceae).

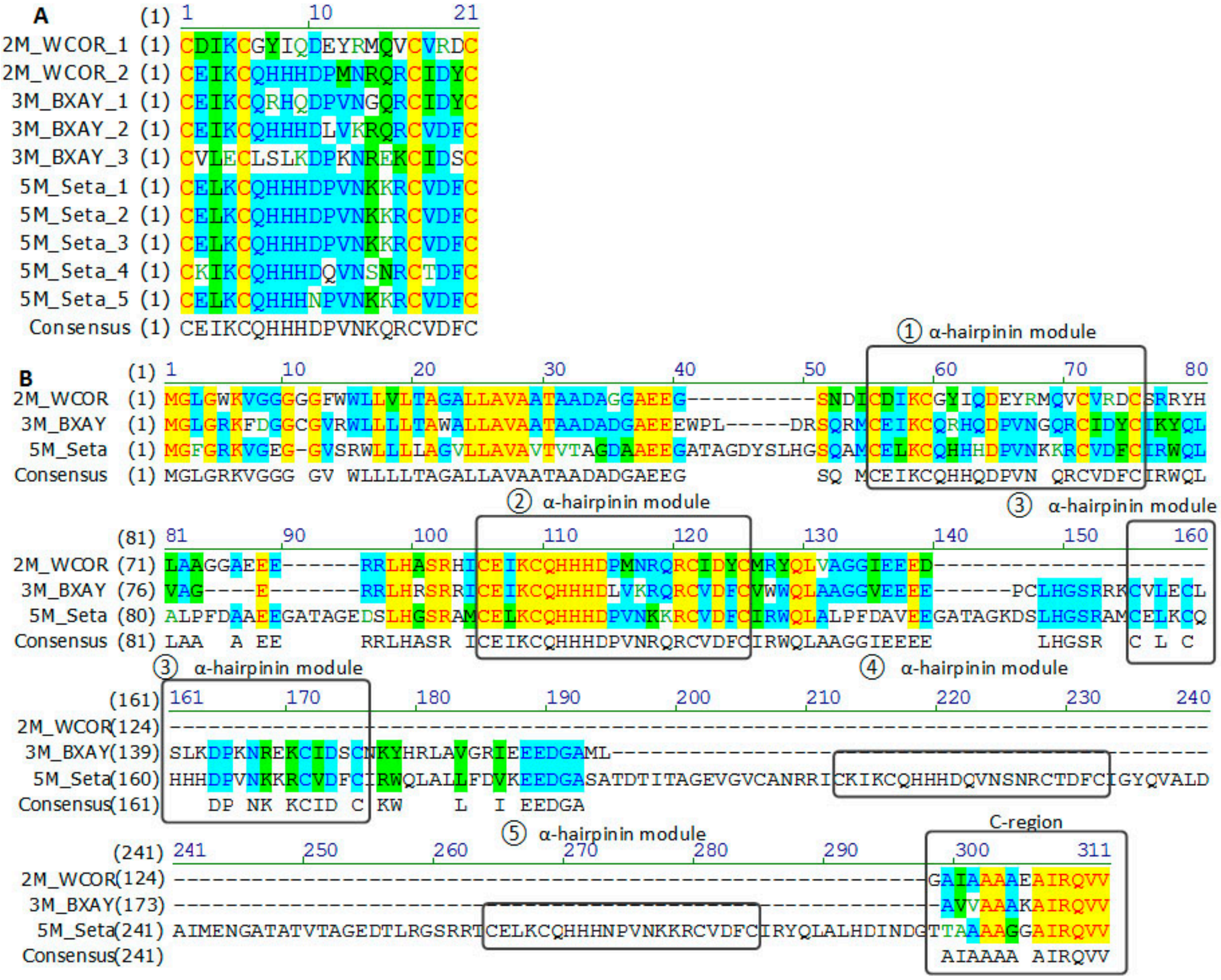

Figure S4. Multiple alignments of α-hairpinin-modules referred to the second class of precursors. (**A**) Multiple alignment of selected α-hairpinin module. (**B**) Multiple alignment of the whole selected α-hairpinin precursors. WCOR, *Thyridolepis multiculmis* (Poaceae); BXAY, *Neurachne minor* (Poaceae), Seta, *Setaria italic* (Poaceae).

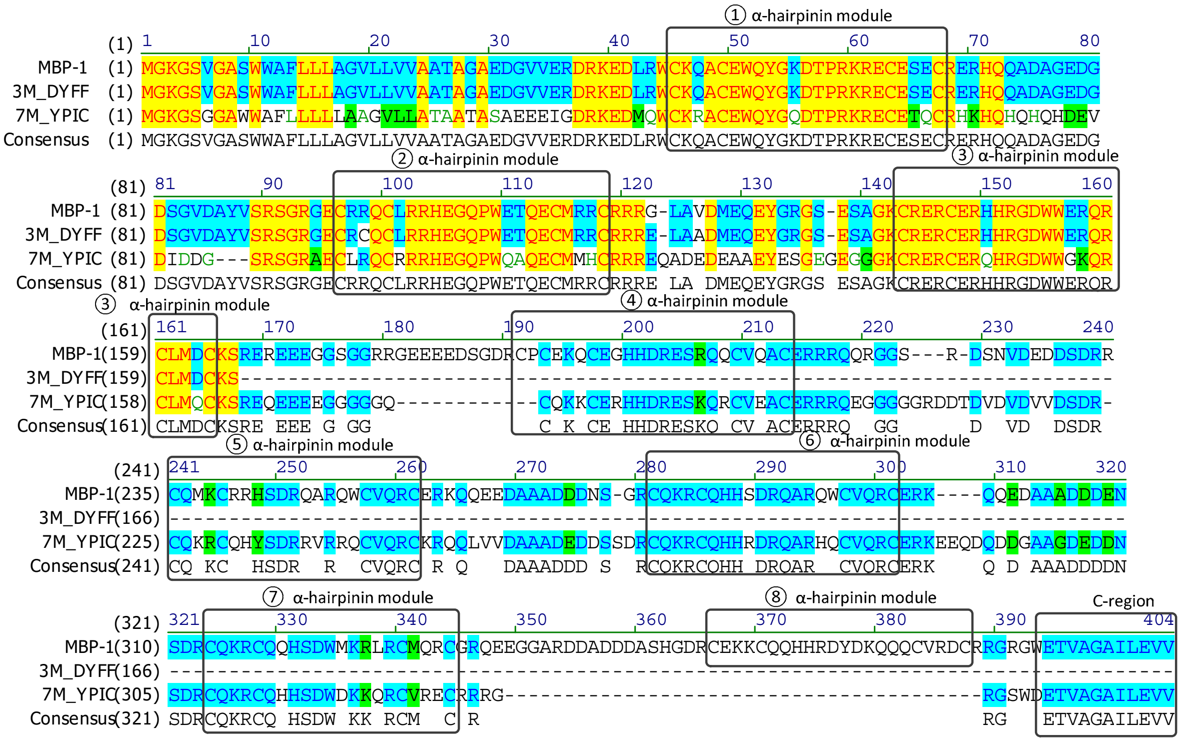

Figure S5. Multiple alignments of selected modular α-hairpinin precursors. MBP-1, precursor of antimicrobial peptide MBP-1(NP_001142639), isolated from *Zea mays* (Poaceae); DYFF, *Pycnanthemum tenuifolium* (Lamiaceae); YPIC, *Microstegium vimineum* (Poaceae).

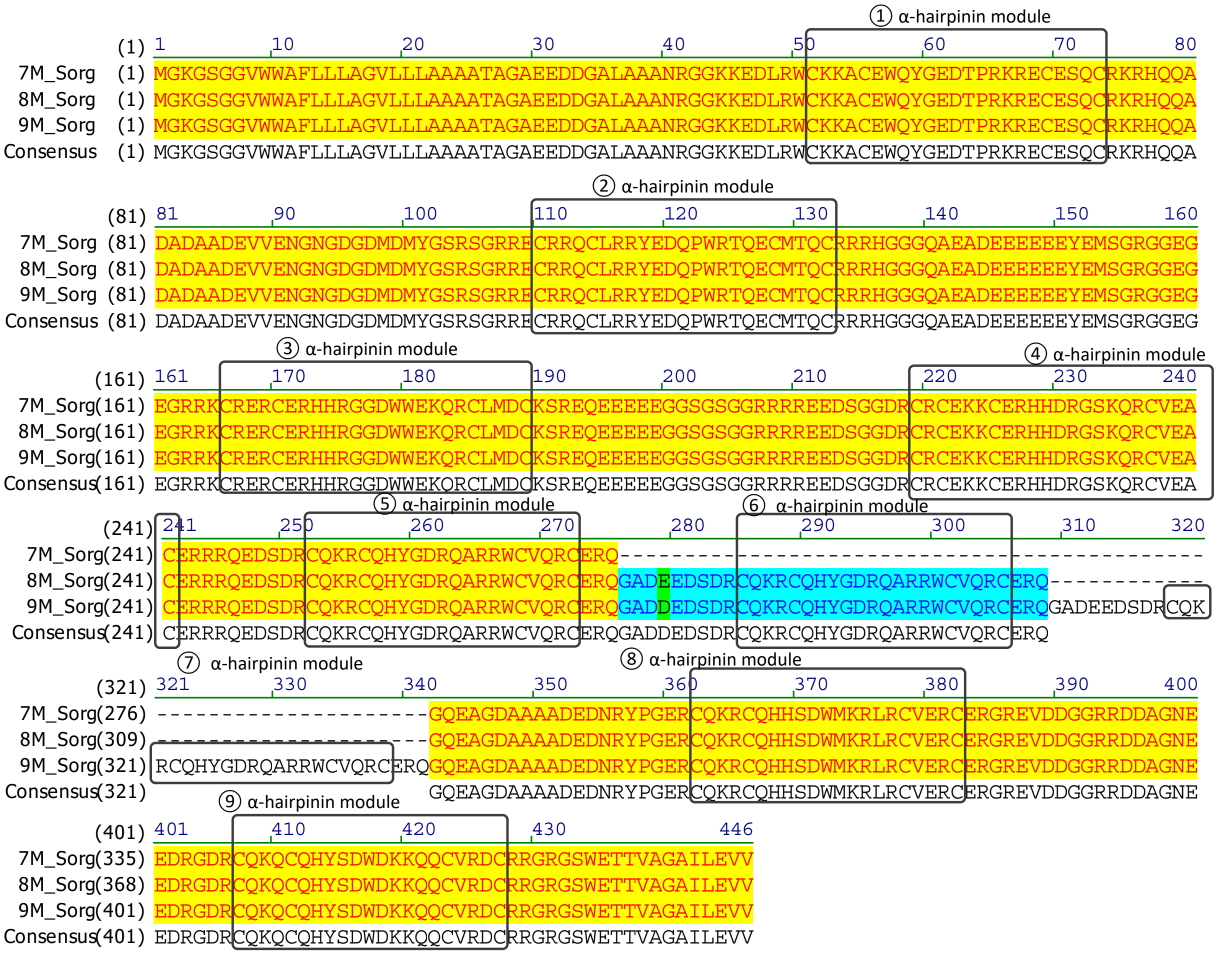

Figure S6. Multiple alignments of modular α-hairpinin precursors found in *Sorghum bicolor*. 7M_Sorg, 8M_Sorg, 9M_Sorg term the precursors with Genbank ID XP_021316825.1, XP_021316824.1 and XP_002449751.1, respectively.
